# Community-based hearing screening for young children using an mHealth service-delivery model

**DOI:** 10.1080/16549716.2018.1467077

**Published:** 2018-05-15

**Authors:** Shouneez Yousuf Hussein, De Wet Swanepoel, Faheema Mahomed, Leigh Biagio de Jager

**Affiliations:** a Department of Speech-Language Pathology and Audiology, University of Pretoria, Pretoria, South Africa; b Department of Speech-Language Pathology and Audiology, University of Pretoria, Pretoria, South Africa

**Keywords:** Smartphone, hearing screening, early childhood development, mobile health, community healthcare workers

## Abstract

**Background**: Hearing loss is one of the most common developmental disorders identifiable at birth with its prevalence increasing throughout school years. However, early detection programs are mostly unavailable in low- and middle-income countries (LMICs) where more than 80% of children with hearing loss reside.

**Objective**: This study investigated the feasibility of a smartphone-based hearing screening program for preschool children operated by community healthcare workers (CHWs) in community-based early childhood development (ECD) centers.

**Method**: Five CHWs were trained to map ECD centers and conduct smartphone-based hearing screenings within a poor community in South Africa over a 12-month period. The hearScreen^TM^ smartphone application employed automated test protocols operating on low-cost smartphones. A cloud-based data management and referral function allowed for remote monitoring for surveillance and follow up.

**Results**: 6424 children (3–6 years) were screened for hearing loss with an overall referral rate of 24.9%. Only 39.4% of these children attended their follow-up appointment at a local clinic, of whom 40.5% referred on their second screening. Logistic regression analysis indicated that age, gender and environmental noise levels (1 kHz) had a significant effect on referral rates (p < 0.05). The quality index reflecting test operator test quality increased during the first few months of testing.

**Conclusion**: Smartphone-based hearing screening can be used by CHWs to detect unidentified children affected by hearing loss within ECD centers. Active noise monitoring, quality indices of test operators and cloud-based data management and referral features of the hearScreen^TM^ application allows for the asynchronous management of hearing screenings and follow-ups.

## Background

Hearing loss is one of the most common developmental disorders identifiable at birth which, if left undetected, has a negative impact on a child’s speech, language, cognitive, educational and socio-emotional development [1,2]. Approximately 0.5 to 5 in every 1000 neonates and infants have congenital, early childhood onset sensorineural or severe-to-profound hearing loss [3]. Hearing loss may lead to developmental delay and difficulty progressing in school if timely and optimal interventions are not provided [3]. These children are therefore at a greater risk for failure and drop-out from school thus placing the children at an economic disadvantage [4]. Even a unilateral hearing loss in children poses significant risk factors such as increased rates of grade failure, the need for additional educational assistance, and perceived behavioral issues in the classroom [5–7].

Unfortunately, there are limited prospects of identifying hearing loss in children, particularly within developing regions such as sub-Saharan Africa where an estimated 6.8 million children suffer from permanent disabling hearing loss [5,8,9]. This may be attributed to the absence of early hearing detection and identification (EHDI) programs due to reasons including limited human resources for ear and hearing care, a lack of appropriate equipment, costs and other health care priorities [10,11]. The World Health Organization (WHO) estimates that there is only one audiologist per 0.5 million to 6.25 million people in the developing world [11], with countries in sub-Saharan Africa presenting with a ratio of one audiologist per 0.8 million people [12].

Community-based hearing programs have been proposed as a way to improve access to ear and hearing care [13]. WHO primary ear and hearing care training manuals recommend that primary health care workers and community healthcare workers (CHWs) in LMICs are trained to stimulate and encourage greater prioritization of prevention, identification and treatment of hearing loss [14]. Prevention or early identification can reduce the negative consequences of a hearing loss, is usually less expensive and can often be implemented at a community level [13,15]. Costs may also be reduced by using innovative technologies using mobile health or mHealth applications [13,16].

With the widespread penetration of 4.92 billion mobile phones worldwide, of which more than 3.74 billion are smartphones, mHealth hearing applications are demonstrating promise to improve access to hearing services in low- and middle-income countries (LMICs) [16–20]. One such mHealth solution, validated in various contexts, is the hearScreen^TM^ solution that allows a low-cost alternative to conventional hearing screening equipment whilst adhering to required acoustic calibration standards [21,22].

The hearScreen^TM^ mHealth solution allows for pre-specified screening protocols with automated sequences to be employed by non-specialist personnel [21,23]. This means generalist healthcare workers or schoolteachers can be trained to operate the device, after which patient-specific data and results collected on the smartphone application can be uploaded to a centralized cloud-based server through cellular networks. This allows for asynchronous point-of-care diagnostics in difficult to reach populations, with cloud-based data management, surveillance and referrals that in turn may reduce the demand placed on already limited professional ear and hearing health human resources in developing countries.

A smartphone-based application may also offer other benefits essential when testing in informal settings, including environmental noise monitoring, quality control indices of test operators and data management [21,22]. The hearScreen^TM^ software employs noise monitoring algorithms which provide operators with real-time feedback on ambient noise levels, thereby providing a guide to minimize the effect of noise levels when testing in varying noise conditions [21,22]. The hearScreen^TM^ automatically retests frequencies where maximum noise levels were exceeded [21,22]. Furthermore, smartphone applications can employ a geotag feature to immediately link patients to their closest hearing health providers or primary healthcare facility with text message notifications.

Pure tone audiometry screening in schools using the hearScreen^TM^ application has demonstrated a low-cost, accurate and efficient asynchronous screening solution that could be facilitated by non-specialist personnel with limited training [22]. However, no systematic clinical validation has been conducted for using this solution to identify younger, more difficult to test preschool children. Early childhood development (ECD) centers are aimed at providing emotional, cognitive and physical development of children from birth to school-going age in addition to a focus on a child’s nutrition, health, psychological and other needs [24]. With the integration of asynchronous, low-cost mHealth technologies, early childhood development centers in LMICs could therefore have the potential to serve as the first point of access to preventative hearing healthcare services for children from underserved populations prior to school entry. Therefore this study set out to determine the feasibility of a low-cost, ECD hearing screening program for preschool children operated by CHWs using an mHealth point-of-care diagnostic and cloud-based data management, surveillance and referral system.

## Methods

### Context

The study was conducted in the community of Mamelodi, City of Tshwane, Gauteng, South Africa. Mamelodi is situated approximately 20 km east of the city. The unofficial population of Mamelodi is currently close to one million. Census indicated 110,703 households within the community of which only 61% are formal dwellings [25].

### Participants

Non-probability purposive sampling was used to select participants. Initially, three CHWs were trained to map ECD centers and conduct hearing screenings. An additional two CHWs joined the project during the last three months to assist with the workload. CHWs were first trained to map ECD centers using the facility-mapping feature of the hearScreen^TM^ application. This feature allowed CHWs to record the name of the ECD facility, geolocation, contact person and number of children enrolled. CHWs were thereafter trained to conduct hearing screenings using the hearScreen^TM^ application. These participants had no formal training on hearing healthcare. Prior to implementation of the project, CHWs received a training session during which they were provided with information regarding ear and hearing healthcare, and its importance, as well as training and hands-on practice with the hearing screening smartphone application.

Two hundred and fifty ECD centers were mapped in the community of Mamelodi East and West. ECD centers (crèches) included both public and private facilities that provided learning and support to children between the ages of three to six years. These ECD centers were often informal in nature and based in the homes of local community members. Once consent was obtained from the principal of the ECD centers to conduct hearing screenings, consent letters were sent to the parents/caregivers. Data was collected over a 12-month period with the exception of 3 vacation periods.

### Equipment

The hearScreen^TM^ smartphone application was initially operated on Samsung Trend Plus (S5301) smartphones (Android OS, 4.0). In July 2016, the Samsung Trend plus smartphones were replaced with Samsung J2 Galaxy smartphones (Andriod OS, 5.1), which operated an upgraded version of the hearScreen^TM^ software. Smartphones were connected to supra-aural Sennheiser HD280 Pro headphones (Sennheiser, Wedemark, Germany). The hearScreen^TM^ calibration function was used to calibrate the headphones according to prescribed standards (ISO 389–1:1998) adhering to equivalent threshold sound pressure levels determined for this headphone [26].

The hearScreen^TM^ application records a quality index of test operators, which gives an objective measure of their screen performance. During each test, a randomized false presentation of a sound is presented. The purpose of this presentation is to determine if the screener correctly records the response by the person tested as ‘no response.’ The hearScreen^TM^ then calculates a quality index on the tests completed by each screener to provide an indication of the reliability of the tests conducted by the test operator. This quality index was monitored throughout the project in order to guide and retrain testers when needed.

Noise levels are also recorded by the smartphone hearing screening application for each child during testing. In order to minimize false-positive results caused by exceeded noise levels, testing was not conducted at 0.5kHz. The hearScreen^TM^ solution has been validated to monitor noise accurately [21]. Data collected by the smartphone was automatically uploaded to a secure cloud-based server through a 3G cellular network.

### Procedures

Once mapping was completed, hearing screenings were only conducted on a set test date if consent was granted by both the ECD center and the parent/caregiver. ECD center staff allocated a room with the least noise possible for testing. Children were provided with a simple explanation and demonstration of what was required of them. The hearing screening application employed automated test protocols. In order to ensure that the child understood what was expected, testing began in the left ear with an initial conditioning tone at 1 kHz at an intensity level of 35 dB HL. Thereafter, a sweep was performed at the test frequencies of 1, 2 and 4 kHz bilaterally at a screening intensity of 25 dB HL [21]. The smartphone microphone measured noise levels in the environment and employed a smart noise-monitoring algorithm that only initiated a rescreen if noise levels exceeded maximum permissible ambient noise levels (MPANLs) when there was no response from a patient. The stimulus was repeated once if the child did not respond at any frequency. Once data was collected for the left ear, the same procedure was repeated in the right ear.

Failure to respond at 25 dB HL at any frequency in any ear constituted an initial fail. In such cases, children were reconditioned and an immediate rescreen was initiated which followed the same procedure [2]. If a child referred the immediate rescreen at the ECD center, he/she was referred to his/her local clinic for a second hearing screening followed by a diagnostic hearing assessment if necessary. This was done by automatically sending a text message notification to parents via the cloud-based server. Additionally, results and test quality were remotely monitored from the cloud-based data management portal. ECD facilities were provided with a summary of results for educational interventions.

### Data analysis

Data were extracted from the cloud-based server to an MS Excel (2011) sheet and analyzed using SPSS v24 (Chicago, Illinois). Referral rates, test times, noise levels and quality indices of testers were analyzed using descriptive statistical measures. Binomial logistic regression analysis was used to determine the effects of age, gender and exceeded MPANLs on referral rates in children, with p < 0.05 used to indicate a significant effect. Frequency distributions were also used to analyze the quality indices of tester.

## Results

A total of 6424 children (3446 females, 2978 males) between the ages of 3 and 6 years were screened at ECD facilities. Initial screen referral rates were 34.8% (Table 1), with no significant difference between left and right ears (p > 0.05, chi-square). A total of 2227 children were rescreened automatically after the initial failed screen, resulting in an overall referral rate of 24.9% varying from 19.6 to 45.8% for children 6 and 3 years of age, respectively. A rescreen was not completed for nine participants due to a tester inadvertently selecting to skip the procedure. Mean test duration, including both initial and rescreen test times for both ears, was 68 seconds (SD 2.8) for participants who passed and 258.5 seconds (SD 251.2) for those who failed.

**Table 1.** Referral rate for smartphone hearing screenings in ECD centers.

	Screening at ECD centers		Second screening at clinics	
	Participants (n)	Referral Rate (%)	Participants (n)	Referral Rate (%)
Initial screen	6424	34.8	617	50.2
Left overall	6424	25.2	617	36
Left 1kHz	6424	18.5	617	27.9
Left 2kHz	6424	14.1	617	19.6
Left 4kHz	6424	9.8	617	18.3
Right overall	6424	26.4	617	39.9
Right 1kHz	6424	21	617	30.6
Right 2kHz	6424	13.7	617	22.2
Right 4kHz	6424	11	617	21.1
Immediate rescreen	2227	70.3	309	80.6
Left overall	2227	52.9	309	59.9
Left 1 kHz	2227	49.2	309	46.9
Left 2 kHz	2227	40.2	309	29.4
Left 4 kHz	2227	31.6	309	32.4
Right overall	2227	57.5	309	68.9
Right 1 kHz	2227	55	309	56.3
Right 2 kHz	2227	41.5	309	40.5
Right 4 kHz	2227	35.4	309	35.3
Overall referral result	6424	24.9	617	40.5

Average noise levels recorded during the initial screen at ECD centers were 44, 41 and 40 dB for 1, 2 and 4kHz respectively. MPANLs were exceeded occasionally during the initial and immediate rescreens conducted at 1, 2 and 4 kHz at both ECD centers and clinics (Table 3).

**Table 2.** Referral rate in children according to gender and age groups.

	Screening at ECD centers		Rescreening at clinics	
	Participants (n)	Referral Rate (%)	Participants (n)	Referral Rate (%)
Gender				
Female	3446	26.9	243	41.2
Male	2978	22.7	374	40.1
Age groups				
3 years	504	45.8	71	62
4 years	1519	30	141	39
5 years	2259	22	195	36.9
6 years	2142	19.6	210	37.6

**Table 3.** Instances where noise levels exceeded MPANLs during smartphone screening.

	Frequencies	1kHz		2kHz		4kHz	
		Left	Right	Left	Right	Left	Right
Screening at ECD centers	Initial Screen (n = 6424)	8.3%	7.2%	0.9%	0.9%	0.9%	0.9%
	Immediate rescreen (n = 2227)	6%	5.7%	0.3%	0.2%	0.2%	0.1%
Second screening at clinics	Initial Screen (n = 617)	6.2%	6.5%	1.5%	1.6%	1.5%	1.5%
	Immediate rescreen (n = 309)	5.5%	7.1%	1.0%	0.6%	0.6%	0.3%

A binomial logistic regression was performed to ascertain the effect of gender, and noise levels at each frequency, as well as age on overall results obtained at the ECD centers. The logistic regression model was statistically significant (χ^2^(8) = 185.412, p < 0.001) and correctly classified 75.1% of the cases. Referral rates were significantly affected by age (p < 0.01; *B*: −0.004; 95% CI lower: 0.996, 95% CI upper: 0.997), gender (p < 0.01; *B*: 0.231; 95% CI lower: 1.122, 95% CI upper: 1.415), and noise levels at 1kHz in the left ear (p < 0.01; *B*: 0.356; 95% CI lower: 1.103, 95% CI upper: 1.847). Females were 1.26 times more likely to fail compared to males, and increasing age was associated with a decreased likelihood of failure (Table 2).

Failures were monitored and referred to their local clinic for a follow-up via the cloud-based data management system. The follow-up constituted a rescreen and diagnostic test if indicated. A total of 617 children attended their follow-up appointments (Table 1), indicating a follow-up return rate of 39.4%. The overall follow-up screen referral rate was 40.5%. The mean test duration recorded was 170.7 seconds (SD 199.3) and 141.5 seconds (SD 188.2) for pass and failure rates respectively.

Quality indices of test operators were monitored throughout the test period. Table 4 displays the increase in quality indices of the first three test operators over a five-month period.

**Table 4.** Quality index of test operators.

		Month 1	Month 2	Month 3	Month 4	Month 5
Tester 1	No. of children screened	92	270	282	142	179
	Quality index (%)	95	96	99	99	100
Tester 2	No. of children screened	71	261	245	178	189
	Quality index (%)	92	81	96	99	99
Tester 3	No. of children screened	58	202	166	100	129
	Quality index (%)	69	90	96	97	100

## Discussion

Performing hearing screenings within preschool aged populations is important to identify hearing health concerns that may interfere with language development and future school success [2]. Within LMICs, ECD centers have the potential to serve as the first point of access to identify these children. This study provides a baseline for the implementation of a low-cost, ECD hearing screening program operated by CHWs using an mHealth point-of-care diagnostic and cloud-based data management and referral system.

The referral rate in the preschool-aged population (3–6 years) using the hearScreen^TM^ application was 24.9%. Studies using conventional pure tone audiometry reported similar referral rates of 21.5% (2–6 years) and 21.3% (3.5–6 years) [27,28]. In contrast, a recent study conducted using the hearScreen^TM^ smartphone application indicated a significantly lower referral rate of 4.3% for older children aged 5 to 7 [21]. Higher referral rates in the current study are likely due to the fact that testing was conducted in a poor community where risk factors such as otitis media are higher, and due to the inclusion of younger children aged three and four years who presented with high referral rates. Results indicated a lower risk of failure in older compared to younger children. A previous study reported similar findings with a decrease in referral rate as the age of children increase [29].

Referral rates of the current study were greatest in children aged 3 years (45.8%) as opposed to older children, which prompted the researchers to discontinue testing this age group. A study by Sideris and Glattke [28] found that children younger than four years were often unable to perform pure tone screening, suggesting that pure tone audiometry requires a higher level of cognitive maturity. Additionally, the incidence of acute otitis media and otitis media with effusion is high in LMICs, with a higher incidence in children between the ages of two and five years, thus adding to a higher referral rate [30–32].

Environmental noise can also have an effect on referral rates, particularly when testing within an ECD setting where noise levels often fluctuate due to the absence of a sound-treated room, children leaving or entering the test environment, testers providing instructions, or groups of children walking past the test room [2,21,29]. A smartphone-based mHealth solution like hearScreen^TM^ utilizes integrated noise monitoring, providing operators with real-time feedback on noise levels to allow testers to minimize noise levels before continuing with tests. Results and corresponding noise levels analyzed on the centralized cloud-based server indicated that only noise levels at 1 kHz had a significant effect on referral rates obtained in comparison to 2 and 4 kHz test frequencies. Previous studies using the hearScreen^TM^ application also reported similar effects when testing at the lower frequency of 1 kHz [21–23].

Increasing screening intensities to 30 dB HL at 1 kHz to compensate for high noise levels in future community-based studies could reduce the incidence of exceeded MPANLs and false-positive results, but may decrease the validity of the screening process as mild losses may be missed [23,29]. The European Consensus Statement on Hearing, Vision, and Speech Screening in Pre-School and School-age Children indicated that although hearing screenings will produce over-referrals, false positives are preferred over false negatives [33].

Gender effects were evident in smartphone hearing screening outcomes, with females more likely to refer than males. Mahomed-Asmail et al. [22] also reported a significantly higher referral rate in school-aged females using conventional screening; however, these gender effects were not evident when using smartphone hearing screening. One possible reason was attributed to hair length or styles in girls that could have affected headphone placement. Further investigations on gender-specific results are needed.

During follow-up appointments at clinics, ECD screening results were initially pulled from the cloud-based data system and analyzed. Thereafter, children received a second rescreen at the clinic. This was done in order to avoid unnecessary diagnostic assessments and to reduce the workload on already strained audiologists. Less than half (40.5%) of the children who failed their ECD screening failed the second screen at the clinic. The referral rate dropped by a further 5% (35.7%) when excluding more difficult to test children aged three to four years. Some influences which may have contributed to the difference in referral rates include ambient noise levels, headphone placement, visual distractions, and examiner instructions and expertise [29].

Mean test durations, including the initial and immediate rescreen of both ears, were 177.8 and 174.3 seconds when testing at ECD centers and clinics respectively. Wu et al. [34], reported a slightly shorter test duration of 149.4 seconds when using a smartphone hearing screening application. Higher test times at ECD centers could be attributed to the longer test times recorded with failure rates (258.5 seconds) in comparison to pass rates (68 seconds) due to the additional time required to recondition the child being rescreened. Furthermore, mean screen times were significantly higher for 3-year-olds when testing at ECD centers (193.4 seconds) and clinics (239.8 seconds). Only initiating a rescreen for failed frequencies during rescreens, instead of repeating the entire screen sequence, may reduce test times.

Although parents were notified of their child’s result via an SMS sent automatically from the cloud-based system, a low follow-up return rate of 39.4% was found. We suspect that this rate was affected by a long waiting period before follow-up appointments, parents changing their mobile phone number and not notifying the ECD center, and difficulties with taking leave from work, which may result in loss of income for informal workers. Other reasons that may account for non-attendance include lack of transportation, fear and uncertainty about the referral clinic, lack of education regarding hearing loss, and a lack of visibility of services [35]. More precise reasons for non-attendance should be investigated in future studies. Incorporating a system to send a second text message reminder three days prior to a child’s appointment may assist in improving follow-up rates [36,37]. Alternatively, immediate onsite hearing assessments could be incorporated into the screening program.

Immediate onsite automated audiometry could motivate parents to attend follow-up appointments by providing an immediate indication of the severity of a hearing problem and thereby also reduce the number of appointments that parents need to attend at clinics [38]. Using onsite automated diagnostic audiometry, facilitated by the same smartphone, could ensure direct referrals for audiological or medical intervention, and may also reduce false positive results [39–41]. In turn, this will improve the cost-effectiveness, feasibility and credibility of the screening program with parents and physicians [29].

Asynchronous cloud-based monitoring and surveillance allowed for quality indices of test operators to be monitored throughout the test period to ensure quality control (Table 4). This guided project managers to provide feedback, additional information and more training to CHWs to ensure reliable test results. High-quality indices of test operators show that CHWs can successfully screen for hearing loss in children. The integrated cloud-based data management system also allowed for advanced features like location-based referrals via text message, reporting and determining follow-up return rates at clinics.

## Conclusion

ECD hearing screening programs using an mHealth point-of-care diagnostics and cloud-based data management and referral systems can be successfully implemented by CHWs within LMICs to identify children prior to school entry. This mHealth model provides a means to improve the cost-effectiveness, quality and efficiency of, and access to, hearing health services in poorer communities, particularly where hearing healthcare providers are unavailable. Quality control features including integrated noise monitoring, quality control indices of test operators and data management allow for asynchronous remote management to ensure reliable testing and to intervene when necessary. Age contributed significantly to high referral rates, suggesting an optimal screening age of five to six years of age. Environmental noise also posed a challenge when testing at the frequency of 1 kHz. Methods to improve the parental follow-up rate should be explored in future studies.

## Paper context

Childhood hearing screening programs within low- and middle-income countries are few, due to limited financial and human resources. This study investigated the feasibility of a low-cost smartphone-based hearing-screening program for preschool children operated by non-specialist healthcare workers. Such programs can successfully aid in prevention, early identification and treatment of disabling hearing loss prior to school entry. Additionally, features such as automated test sequences, integrated noise monitoring and cloud-based data management allow for asynchronous point-of-care diagnostics in difficult to reach populations.
